# Thermal Evaluation of Silica-Based Insulated Magnet Wires from the Sol–Gel Process

**DOI:** 10.3390/gels9080619

**Published:** 2023-07-31

**Authors:** Giovana Pereira dos Santos Lima, Sonia Ait-Amar, Gabriel Velu, Philippe Frezel, Abdelhamid Boudiba, Soumaya Lafqir, Arnaud Nicolay, Pierre-yves Herze, Mireille Poelman

**Affiliations:** 1 UR 4025, Laboratoire Systèmes Electrotechniques et Environnement (LSEE), Univ. Artois, 62400 Béthune, France; sonia.aitamar@univ-artois.fr; 2 Green Isolight International, 62113 Labourse, France; giifrezel@free.fr; 3 Materia Nova, ASBL, Parc Initialis, Av. Nicolas Copernic, 7000 Mons, Belgium; abdelhamid.boudiba@materianova.be (A.B.); soumaya.lafqir@materianova.be (S.L.); mireille.poelman@materianova.be (M.P.); 4 Esix Surface Technologies, 7000 Mons, Belgium; arnaud.nicolay@esix.eu (A.N.); pierre-yves.herze@esix.eu (P.-y.H.)

**Keywords:** Sol–gel, magnet wire, enamelling, extrusion, tangent delta, thermal aging, dielectric, thermal index, TGA, SEM, FTIR

## Abstract

The conventional enameling process used in the fabrication of magnet wires requires harmful processes and products. The target of the industry in the actual context of electrification is to increase the electrical machines’ efficiency. Indeed, the electrical insulation systems (EIS) of an electrical machine undergo various environmental constraints that can shorten their lifespans. Consequently, aspects of the insulation need to be improved, such as its thermal resistance. One of the challenges is to implement sustainable technology without losing performance. This work consists of the thermal performance evaluation of new magnet wires insulated by three types of composites of silica-based solution from the Sol–gel process and amorphous polyamide-imide (PAI). These composite coats are overcoated by an extruded thermoplastic resin with and without fillers. Different types of insulation are tested and compared to determine the better configuration. Thermogravimetric analysis (TGA), Fourier transform infrared spectroscopy (FTIR) analysis, scanning electron microscopy (SEM) analysis, curing characteristics by tangent delta curve, and thermal-aging tests at three temperatures were carried out on the different EIS systems. Dielectric measurements were made between thermal-aging cycles. Their basic mechanical, electrical, and thermal characteristics are promising: the cut-through temperature is situated above 430 °C, their breakdown voltage values are between 5 kV and 9 kV (grade 3), and a good adhesion (overcoming more than 140 turns on a peel test). The thermal-aging results have been consistent with the TGA analysis results. The thermal index following the IEC standards was estimated for the selected EIS, which would have the main basic characteristics of a magnet wire of 200 class; moreover, it would be a greener enameled wire compared to the conventional one.

## 1. Introduction

Classical wires frequently used in electrical machine winding applications are composed of poliesterimide (PEI) enamel overcoated with polyamide-imide (PAI), having a thermal class of 200 °C [1]. Hybrid (inorganic–organic) materials and coatings using the Sol–gel process can achieve an interesting performance and have been employed in different application sectors like the biomedical, energy, and metallurgy sectors, among others. Some materials have already been tested in these areas, such as the following:

- Electrical insulation film of silica–alumina composite coat on a steel substrate. The results of this work show good dielectric strength, comparable to high-voltage insulation porcelains [2].
- The Sol–gel coating has also been studied as an option for high-temperature electrical insulation systems (EIS) [3].
- Materials for printed electronic system applications, such as dielectric layers in thin-film transistors [4].

In the case of this work, this technology is evaluated in its electrical machine winding application. 

Previous works published within the framework of the Hi-Ecowire project [5] have demonstrated the possibility of integrating silica-based materials from the Sol–gel process into the insulation configuration of magnet wires [6,7]. The Sol–gel coating process is a method that was first used in an insulation configuration consisting of a hybrid silica enamel covered with an extruded resin [5,8,9]. 

The big challenge is achieving a better compromise between all the properties necessary for a magnet wire, according to international standards. Several properties are necessary for the magnet wires used in winding electrical machines. The following examples are some of them which are shown in this proposed paper [10]:

- Winding manufacturing involves many rapid operations which can implicate insulation damage. The flexibility and the adhesion of the EIS are the key mechanical properties for avoiding failure from the manufacturing insertion of the winding and during the life service of the electrical machine.
- The EIS must also support the electrical field created when the machine is working. The insulation thickness and the material that composes the insulation must be chosen for withstanding the voltage between turns. It is important to know the amplitude of voltage which causes electrical breakdown and the voltage that accelerates the aging of the EIS. The dielectric strength and the partial discharge inception voltage (PDIV) are some of the electrical properties evaluated for the EIS.
- The current passing through the conductor causes the heat of the winding, and the temperature can achieve a critical level causing premature aging of the EIS. The excessive thermal exposure induces an accelerated failure of the EIS, and damage to the electrical machine. Many properties can improve the performance of the insulation to resist thermal exposure, especially the material characteristic of the glass-transition temperature.

Past works have shown some poor mechanical properties of silica-based materials produced by the Sol–gel process, despite their good thermal characteristics [11]. The past works carried out within the Hi-Ecowire framework have shown some limitations of mechanical properties required for magnet wires, when higher concentrations of silica-based Sol–gel composite were integrated into polyamide-imide (PAI) solution [5,8,9]. To deeply exploit this technique in the case of this paper, three types of silica-based solutions from the Sol–gel process in optimized concentrations are evaluated with a thermoplastic polymer extruded on a topcoat. The latter has also shown a contribution to the general properties of those wires. The main aims are to exploit the possible combinations of this hybrid technology and identify the one that best responds to general characterization tests, principally focusing on maximizing thermal endurance. 

This paper reports a continuation of the study made on previous work [6]. The main points of these studies are the following ones:

- The composition of the silica-based solutions studied: The work [6] has exploited different polymers with and without silica-based solutions on the enameling coat, and also the firsts configurations of silica–PAI composite from the Sol–gel process studied in different concentrations. In the case of this paper, the same type of extruded resin tested on past work is used, but the silica-based solution chosen for the magnet wires evaluated in this paper is composed of only one type of polymer (PAI), and it is made from three different Sol–gel solutions with specific concentrations. The difference between them is observed from the tests results presented in this paper.
- The method of the magnet EIS analysis: The basic characterization of magnet wires is executed on both works to validate the basic properties of the wires studied before any thermal analysis. Some of the test’s evaluations are carried out only for the wires studied in this paper, such as the thermogravimetric analysis (TGA), scanning electron microscopy (SEM) and the thermal index estimation.

## 2. Results and Discussion

The results are presented in three principal parts:

- Surface and cross-section morphology observation coupled with EDX measurement obtained on the enameled wire (coated copper wires with the studied EIS), FTIR and TGA analyses;
- The general properties of the wire characterization from the mechanical and electrical tests;
- The results from the thermal tests carried out on the insulated wires and the thermal tests caried out in the material insulation.

### 2.1. Materials Analysis 

#### 2.1.1. Surface and Cross-Section Morphology Observation Coupled with EDX Measurements 

**Figure 1:**
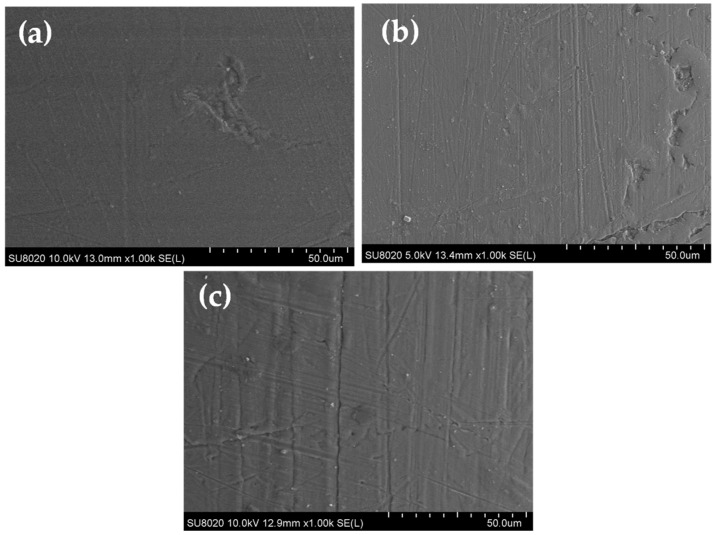
SEM images on the top-surface of coated copper wires with pure extruded resin: (**a**) EIS Sol–gel 1, (**b**) EIS with Sol–gel 2 and (**c**) EIS with Sol–gel 3.

**Figure 2:**
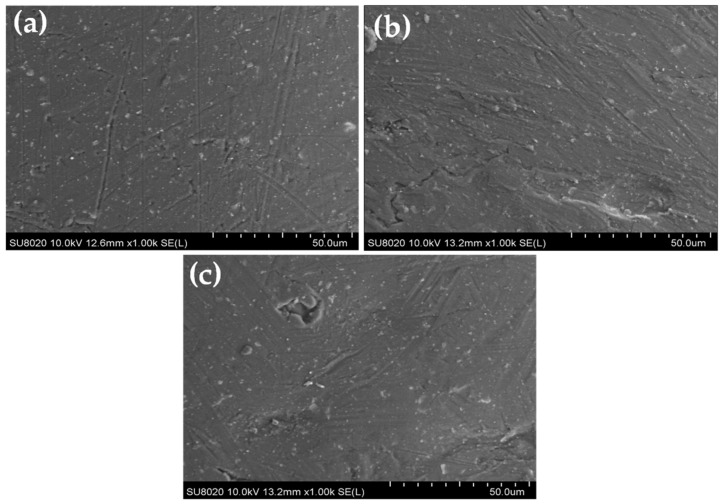
SEM images on the top surface of coated copper wires with filled extruded resin: (**a**) EIS Sol–gel 1, (**b**) EIS with Sol–gel 2 and (**c**) EIS with Sol–gel 3.

The SEM images obtained on the final wires (coated copper wires with the studied EIS) are shown from (a) to (c) in Figure 1 and Figure 2. 

The top surface images reveal a smooth surface and compact morphology for all samples overcoated with pure thermoplastic extruded resins (Figure 1). While the samples with filled thermoplastics EIS (Figure 2) show a top surface morphology with slightly lesser smoothness compared to the ones without fillers. The observed behavior can be explained by the effect of fillers creating small agglomerations within the pure resin.

**Figure 3:**
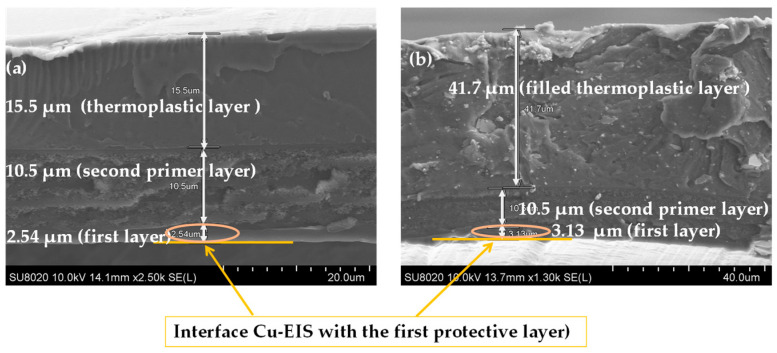
SEM images obtained on the cross-section of coated copper wires EIS Sol–gel 1 (**a**) with pure polymer extruded topcoat and (**b**) with filled extruded polymer.

**Figure 4:**
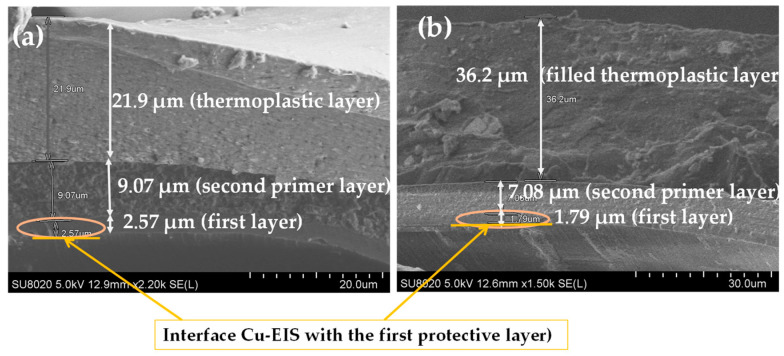
SEM images obtained on the cross-section of coated copper wires EIS Sol–gel 2 (**a**) with pure polymer extruded topcoat and (**b**) with filled extruded polymer.

**Figure 5:**
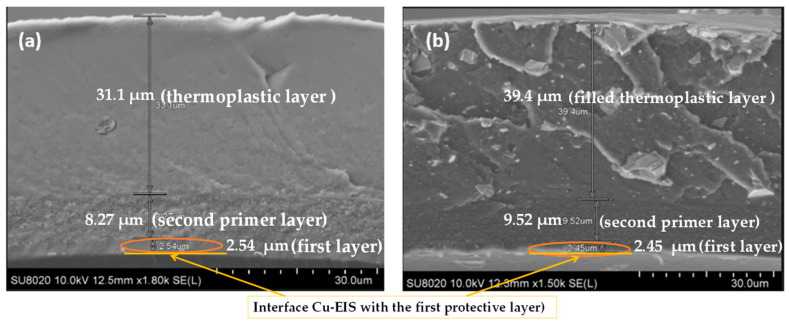
SEM images obtained on the cross-section of coated copper wires EIS Sol–gel 3 (**a**) with pure polymer extruded topcoat and (**b**) with filled extruded polymer.

The SEM images on the cross-section of enameled wires with the EIS system were performed to observe the thickness, architecture of the structure, and the distribution of silica into hybrid composites (the second layer) as well as the filler in the thermoplastic extruded resins. The images carried out on the EIS systems are shown in Figure 3, Figure 4 and Figure 5. The images show the three different layers and the creation of well-dispersed silica particles into the PAI matrix in the dry film with particle sizes in the range of a few hundred nanometers. 

The thickness of each layer is well defined. For example, the wires with EIS Sol–gel 1 give a thickness of the PAI layer of 2.54 µm, PAI/Sol–gel layer of 10.5 µm and thermoplastic extruded polymer topcoat of 15.5 µm, resulting in a total diameter increase of 57.08 µm. In the meantime, the EIS with the Sol–gel 1 overcoat with the same thermoplastic extruded polymer with fillers gives more thickness (41 µm of the topcoat extrusion layer). The wires with EIS Sol–gel 2 give a thickness of the PAI layer of 2.57 µm, PAI/Sol–gel layer of 9.07 µm, and thermoplastic extruded polymer topcoat of 21.9 µm. The EIS with a Sol–gel 2 overcoated with the same thermoplastic extruded polymer with fillers gives a thickness of PAI layer of 2.57 µm, PAI/Sol–gel layer of 9.07 µm and filled thermoplastic topcoat extrusion layer of 36.2 µm. 

The wires with EIS Sol–gel 3 give a thickness of PAI layer of 2.54 µm, PAI/Sol–gel layer of 8.87 µm and thermoplastic extruded polymer topcoat of 31.1 µm. The EIS with a Sol–gel 3 overcoat with the same thermoplastic extruded polymer with fillers gives a thickness of the PAI layer of 2.45 µm, PAI/Sol–gel layer of 9.52 µm and filled thermoplastic topcoat extrusion layer of 39.4 µm.

**Figure 6:**
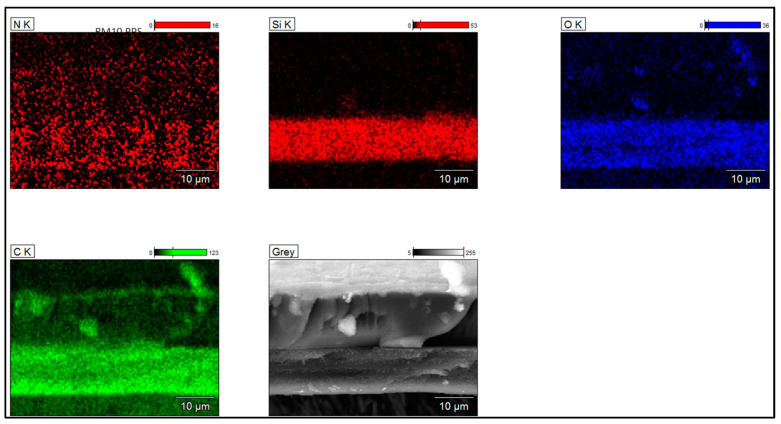
SEM-FEG images coupled with EDX analyses obtained from EIS system (images recorded on the cross-section of enameled wire) showing the dispersion of silica in PAI obtained from Sol–gel 1 (primer layer about 10 µm dry thickness).

**Figure 7:**
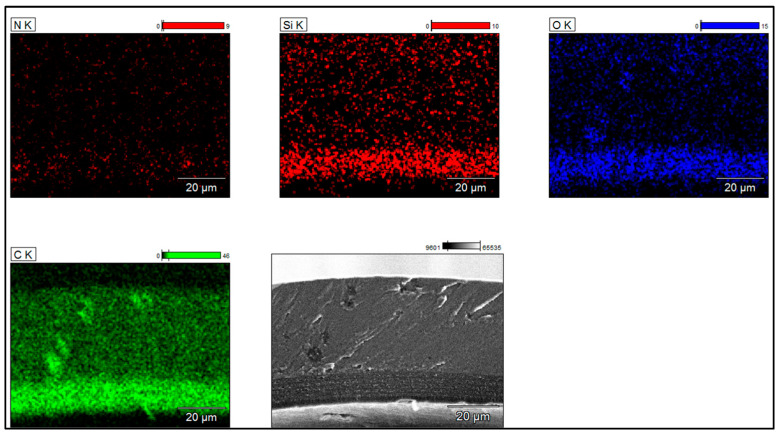
SEM-FEG images coupled with EDX analyses obtained from EIS system (images recorded on the cross-section of enameled wire), showing the dispersion of silica in PAI obtained from Sol–gel 2 (primer layer about 10 µm dry thickness).

**Figure 8:**
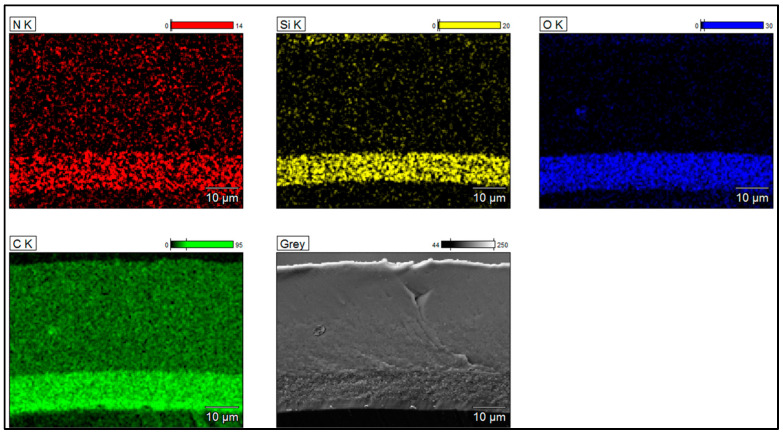
SEM-FEG images coupled with EDX analyses obtained from EIS system (images recorded on the cross-section of enameled wire) showing the dispersion of silica in PAI obtained from Sol–gel 3 (primer layer about 10 µm dry thickness).

To determine the silica dispersion, grain size, and morphology in the primer layers, SEM images coupled with EDX analyses were collected from the samples on the cross-section zones. The EIS systems obtained from the three types of Sol–gel processes are shown in Figure 6, Figure 7 and Figure 8. The silica distribution into PAI polymer chains (represented by Si and O elements and evidenced by FTIR analysis) seems homogeneous and well dispersed into the primer layer (hybrid silica–PAI layers) for each enameling pass with particle size in the nanometric scale (about 250 nm for all EIS systems with Sol–gel 1, 2 and 3). At some zone (primer layer), the O, N, and C elements represent PAI polymer chains.

#### 2.1.2. Thermal Properties from TGA Analysis

The TGA analyses were performed on the samples of the different EIS configurations under air, from RT (25 °C) to 1000 °C (Heating rate 10 °C/min). The composites (dried EIS) were collected directly from the coated wires (about 10 mg of each sample was used for the analyses). 

The parameters obtained from the TGA analyses on the different EIS configurations are summarized in Table 1. 

By assuming that the residue part, from TGA analysis, corresponds to the mineral part introduced by the Sol–gel process in the EIS systems (unfilled EIS), the silica content of the dried coatings (full EIS system, including the three layers, PAI + PAI/Sol–gel + Thermoplastic resin) was 5.68%, 4.38%, and 5.22% at 950 °C for EIS Sol–gel 1, EIS Sol–gel 2 and EIS Sol–gel 3, respectively.

The mineral part (residual) for the dried-coating-filled thermoplastic (PAI + PAI/Sol–gel + filled extruded polymer) was 27.22%, 24.37%, and 23.32% for EIS Sol–gel 1, EIS Sol–gel 2 and EIS Sol–gel 3, respectively. 

**Figure 9:**
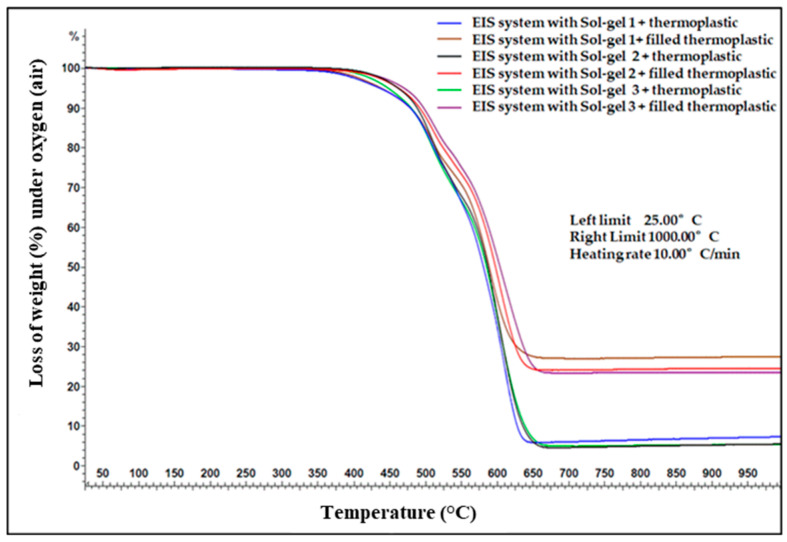
TGA analysis on the different silica gels and composites.

**Figure 10:**
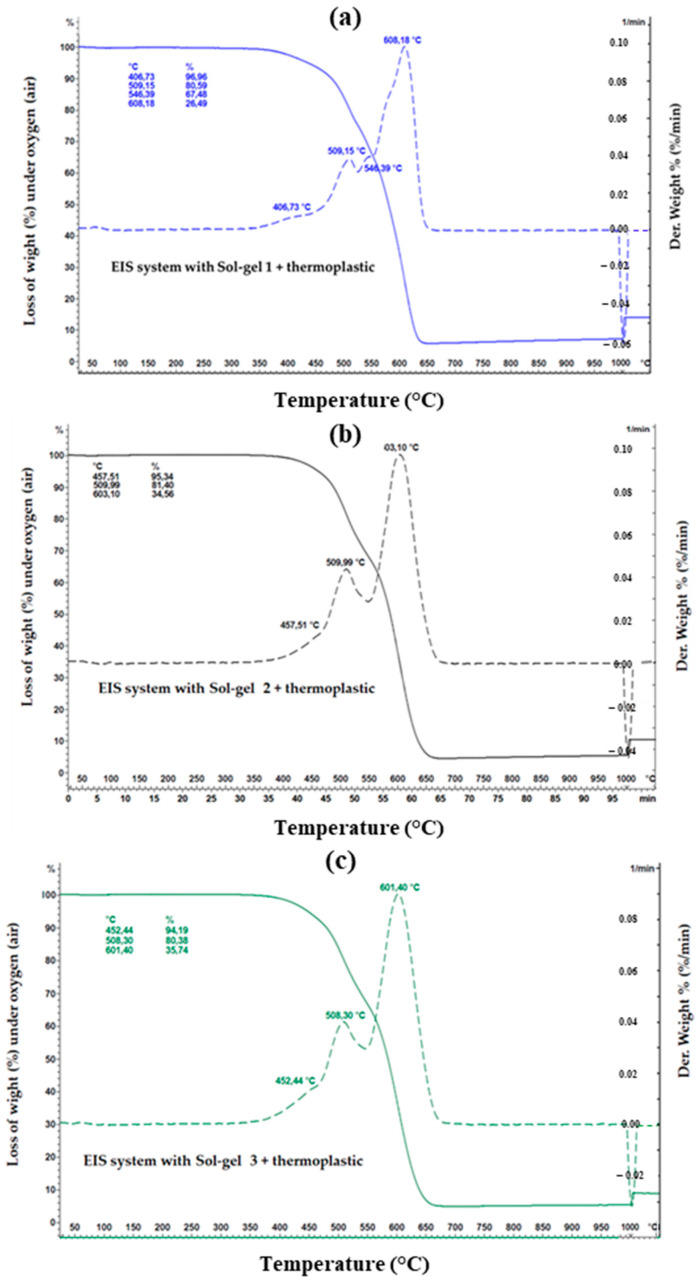
TGA measurement on the EIS systems with thermoplastic topcoat, and each type of Sol–gel from (**a**–**c**).

**Figure 11:**
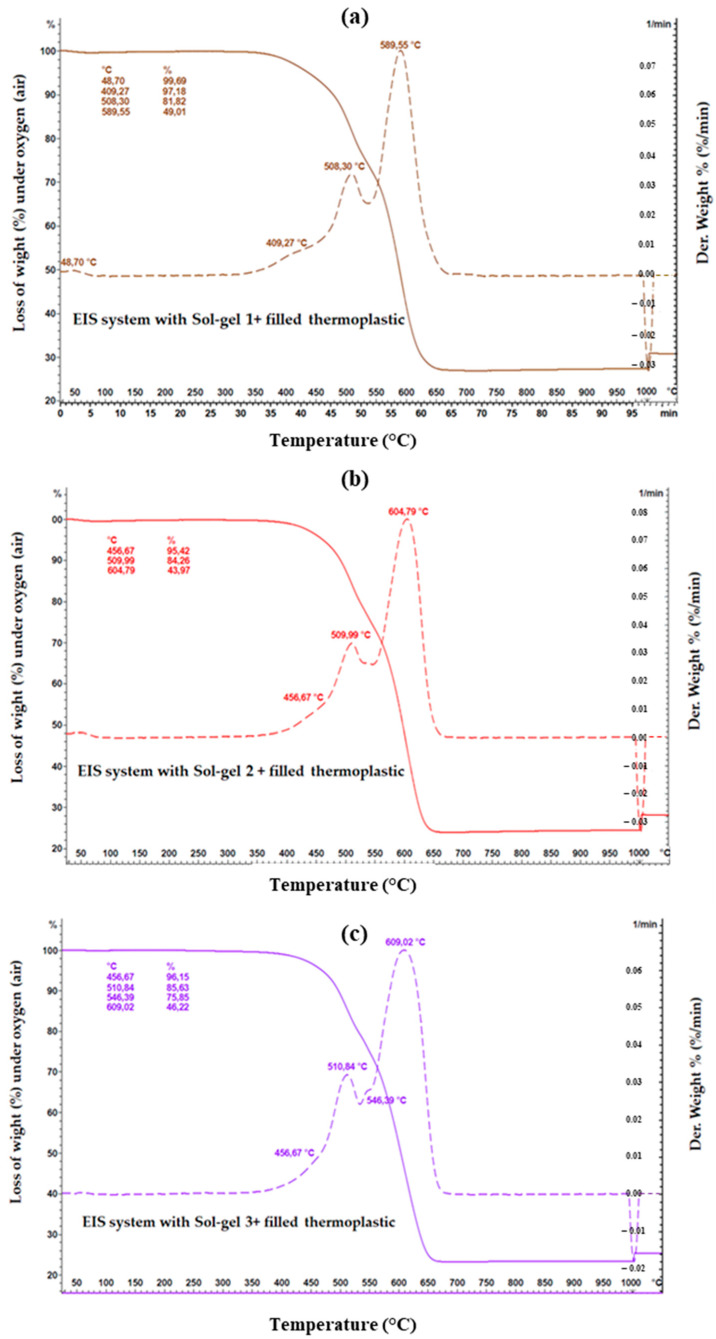
TGA measurement on the EIS systems with filled thermoplastic topcoat, and each type of Sol–gel from (**a**–**c**).

The TGA curves are shown in Figure 9, Figure 10 and Figure 11. It can be observed that between 25 °C and 350 °C, all EIS systems have insignificant mass loss ≤1% regardless of the air atmosphere (oxygen).

Starting from 400 °C, the weight loss (mass loss) starts decreasing, indicating the start of the thermal degradation process.

For the Sol–gel 1 unfilled thermoplastic, three decomposition steps are visible. The first decomposition step is at 406.73 °C with a mass loss of 3.04%. The second decomposition step is split at 509.15 and 546.39 °C with a mass loss of 16.37 and 13.11%. The first and second decomposition steps are related to pyrolytic fragmentation. The third decomposition step at 608.18 °C with a mass loss of 40.99% is related to the carbonization process. The sum of the mass loss of Sol–gel 1 is 73.51%. In the case of Sol–gel 1-filled thermoplastic, four decomposition steps are visible. The moisture content is responsible for the first weight loss (0.31%) at 48.70 °C. The second decomposition step at 409.27 with a mass loss of 2.51%, and the third decomposition step at 508.30 °C with a mass loss of 5.36% are related to pyrolytic fragmentation. The last decomposition step at 589.55 °C with a mass loss of 42.81% is related to the carbonization process.

In the EIS system with Sol–gel 2 unfilled thermoplastic, three decomposition steps are visible. The first decomposition step at 457.51 °C with a mass loss of 4.66% and the second decomposition step at 509.99 °C with a mass loss of 13.94% are related to pyrolytic fragmentation. The third decomposition step at 603.10 °C with a mass loss of 46.84% is related to the carbonization process. In the EIS system with Sol–gel-2-filled thermoplastic, three decomposition steps are visible. The first decomposition step is at 456.67 °C with a mass loss of 4.58 and the second decomposition step at 509.99 °C with a mass loss of 11.16% are related to pyrolytic fragmentation. The third decomposition step at 604.79 °C with a mass loss of 40.29 is related to the carbonization process.

In the EIS system with Sol–gel-3 unfilled thermoplastic, three decomposition steps are visible. The first decomposition step is at 452.44 °C with a mass loss of 5.81 and the second decomposition step is at 508.30 °C with a mass loss of 13.81% are related to pyrolytic fragmentation. The third decomposition step at 601.40 °C with a mass loss of 44.64 is related to the carbonization process. In the EIS system with Sol–gel-3-filled thermoplastic, three decomposition steps are visible. The first decomposition step is at 456.67 °C with a mass loss of 3.85. and the second decomposition step is split at 510.84 and 546.39 °C with mass losses of 10.52 and 9.78%. The first and second decomposition steps are related to pyrolytic fragmentation. The third decomposition step at 609.02 °C with a mass loss of 29.63% is related to the carbonization process.

#### 2.1.3. FTIR Analyses 

**Figure 12:**
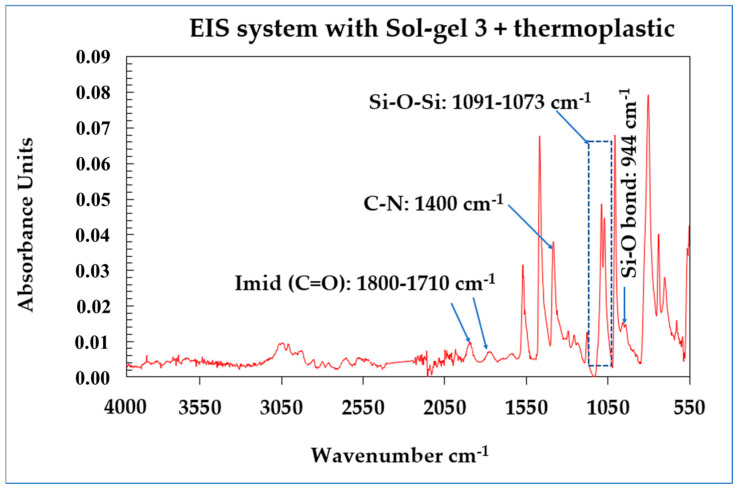
FTIR analysis carried out on the magnet wires with EIS system Sol–gel 3 showing the correlation with EDX/SEM analysis (dispersion of silica particles into PAI primer layer from the Sol–gel process).

The FTIR analysis was measured in the range of 4000–550 cm^−1^ on the enamel wires (coated copper wires with the different EIS systems). The behavior of the unfilled thermoplastic EIS systems (or filled thermoplastic EIS systems) was similar and evidenced the formation of a network of Si-O-Si in the PAI matrices. By correlating the SEM-EDX with FTIR analyses, the conversion of Si-OH (hydrolyzed groups obtained from the starting sols named Sol–gel 1, Sol–gel 2 and Sol–gel 3) to silica (polycondensation state gel Si-O-Si) was evidenced. The Sol–gel process gives a homogeneous dispersion of silica into the PAI matrices. From the FTIR analysis shown in Figure 12, the absence of a broad band between 3000 and 3500 cm^−1^ indicates the absence of OH groups and the full polycondensation of Si-OH to the Si-O-Si network. The character of the asymmetric stretching mode vibration of the Si-O-Si group was observed as a doublet at intense peaks in the range of 1091–1073 cm^−1^, confirming the formation of network Si-O-Si [12,13,14]. From the PAI signature, the amide C=O region is observed around 1710 cm^−1^ and, at 1774 cm^−1^, the absorbance is associated with the imide carbonyl band, and the absorption band for symmetric stretching of C-N was observed at 1385–1363 cm^−1^.

### 2.2. General Mechanical and Electrical Properties of the Insulated Wires

Table 2 presents the results obtained from the mechanical and electrical tests. 

#### 2.2.1. Mechanical Properties

**Figure 13:**
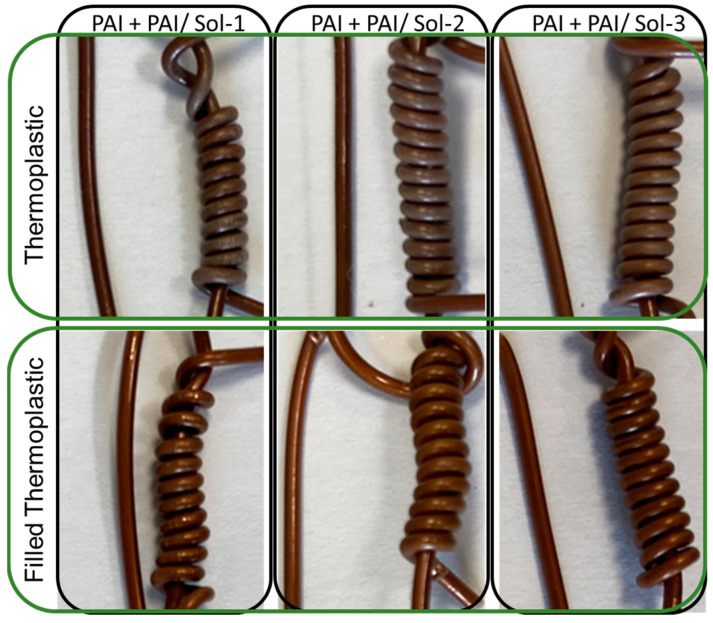
Aspect of the EIS during the flexibility test.

Regarding the flexibility performances, the following conclusions can be drawn:

- The wires composed of the solution Sol–gel 1 (30%)/PAI (70%) showed the presence of cracks when they were wound around their diameter. This solution presents a particularity compared to the others due to the type of Sol–gel and its concentration since, for Sol–gel 1, 30% of the solution is miscible in PAI. The miscibility of this type of Sol–gel is limited to 50%. This is not the case for Sol–gel solutions 2 and 3. However, this higher concentration is possibly a factor that affects the flexibility properties, as already observed in studies at different concentrations [5,8,9].
- The other wires composed of Sol–gel 2 and 3 showed an acceptable appearance; no cracks were visible in the flexibility test. However, the configurations with the filled resin showed a lighter color when wrapped around its diameter. The illustration of Figure 13 show the aspect of each sample next to a piece of wire, to compare the color difference. The standard does not consider the color change as a flexibility problem, as it does the presence of cracks.

Regarding the adhesion performance, the following conclusions can be drawn:

- All the EIS results are satisfactory for the snap tensile test.
- The requirement for a conventional PEI/PAI class 200 wire is K = 110 mm at least, for the peel test according to IEC 60317-13 [1], which corresponds to 115 turns for a wire of diameter 0.95 mm. The peel test shows variations for each type of wire, but all configurations have several turns above 115, which is satisfactory for this test.
- The PAI + PAI/Sol–gel 2 + Filled thermoplastic wires showed a higher performance in the peel test.

#### 2.2.2. Electrical Properties

The variation in thickness between the wires resulted in two different wire grades. This influenced the wire’s electrical properties.

In terms of dielectric breakdown, IEC 60317-0-1 [15] defines a minimum breakdown voltage of 5000 V for 0.95 mm conductor diameter wires for grade 2 and 7600 V for grade 3. From the average breakdown voltage values collected from each insulating configuration, the following conclusions can be drawn:

- Grade 2 wires composed of the non-filled extruded wire present a breakdown voltage that passes the minimum value required by the standard.
- Among the grade 3 wires, the only one that does not have a breakdown voltage higher than the one required by the standard is the PAI + PAI/Sol–gel 2 + Filled thermoplastic, despite its advantage in thickness.
- The filled extruded EIS systems have demonstrated lower values of breakdown voltage compared with the non-filled ones.
- The most advantageous configurations are composed of PAI + PAI/Sol–gel 2 + Thermoplastic.
- The different Sol–gel solutions have not shown a significant influence on the electrical breakdown voltage of the whole system.

### 2.3. Thermal Evaluation Results of the Insulated Wires

Table 3 presents the characteristics obtained by the first thermal group of tests evaluation of the insulated wires.

Regarding the cut-through property, the following conclusions can be drawn:

- The wires insulated with Sol–gel 1 showed the highest cut-through temperature. The presence of Sol–gel 2 or 3 did not show a variation in this property.
- The wires extruded with filled thermoplastic resin did not show a significant difference when compared to the unfilled one.
- Regarding the delta tangent characteristic, the following can be concluded:
- The results of the delta tangent peak temperature show higher values for Sol–gel-1-based compounds in the enamel layer.
- The filled insulated wires compared to the unfilled ones also show an advantage of almost 30 °C.
- The tangent delta characteristic shows the contribution of the filled and Sol–gel types.

**Figure 14:**
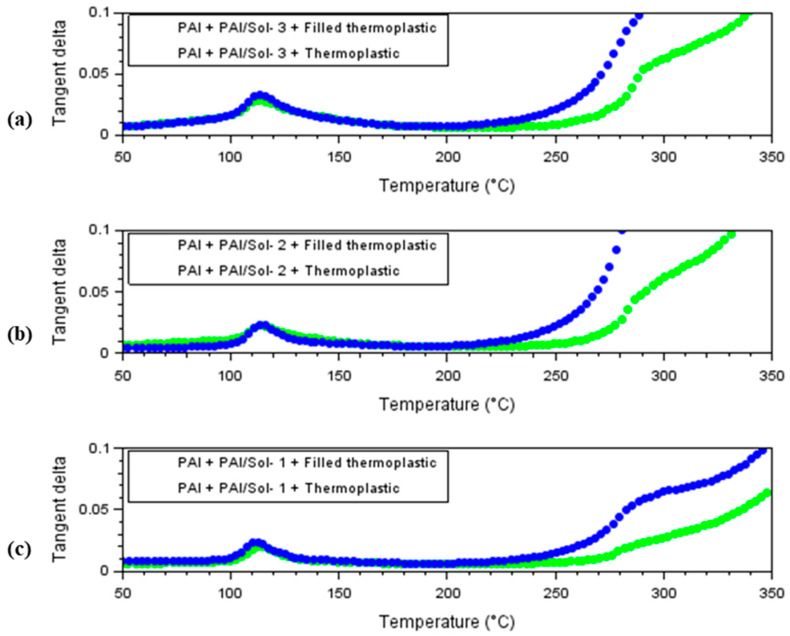
Delta tangent characteristic of extruded enameled wires compound: (**a**) Sol–gel 1, (**b**) Sol–gel 2, (**c**) Sol–gel 3.

To understand the contributions of each PAI/Sol–gel composite, the results in this section are compared to previous works [6]. Their enameled layer is only made of the neat PAI polymer, and the extruded layer is composed of the same resin as those analyzed here: thermoplastic resin and thermoplastic resin filled with the same percentage (25%) of mineral fillers. According to the results from past work [6], the delta tangent peak temperatures are as follows:

- For PAI + Thermoplastic resin, 101 °C and 240 °C;
- For the PAI + Filled thermoplastic resin, 101 °C and 246 °C;
- The first tangent delta peak around 100 °C corresponds to a particular characteristic of thermoplastic resin [16,17]. DMA analyses to characterize the thermoplastic resin consulted in the literature show that, close to 100 °C, there is a phenomenon of relaxation of the rigid amorphous part and an increase in its crystallinity [16,17]. According to the delta tangent curves presented in Figure 14, all six types of wires, including those with thermoplastic resin reinforced with mineral fillers, are characterized by this first delta tangent peak.

The second temperature is influenced by the enamel layer. For the configurations of the neat thermoplastic resin, for example, the following can be said:

- The wire that contains the PAI/Sol–gel 1 composite in enamel has very high temperatures (272.9 and 347.2 °C) that indicate a thermal advantage over the others.
- The PAI/Sol–gel 2 and PAI/Sol–gel 3 composites have very similar peak temperatures (around 270 °C), and it is not possible to identify which one has a thermal advantage over the other.
- The three PAI/Sol–gel composites have between 20 and 30 °C advantage over the PAI + Thermoplastic, especially the PAI/Sol–gel 1 composite.

For the filled resins, the device detects one more tangent compared to the unfilled ones. This reinforces the hypothesis that the latter delta tangent temperature is related to the filled. However, for PAI + Thermoplastic and PAI + Filled thermoplastic, this extra tangent was not identified, so one of the temperatures may be related to the silica particles in the Sol–gel composites.

Knowing that the PAI + Thermoplastic resin and PAI + Filled thermoplastic is manufactured under the same conditions, it is still possible to have variations since they are not manufactured at the same time as the wires with Sol–gel composites. The comparisons made do not consider these possible variations.

The six EIS configurations of this work were aged at the same conditions for the three temperatures presented in Table 4. Regarding the thermal-aging lifetimes, the following conclusions can be drawn:

- At the higher temperature of 270 °C, the lifetimes are equivalent except for the PAI + PAI/ Sol–gel 2 + Filled thermoplastic resin, which obtained a slightly lower lifetime.
- At 250 °C, the lifetimes start to vary between the configurations tested. The filled extruded wires held one cycle longer than the wires extruded with unfilled resin. From this aging temperature test, the positive effect of mineral filler on the extruded resin can be seen. The different Sol–gel composites do not show any influence on this test.
- At the lower temperature of 240 °C, the first cycle was 720 h, and most of the wire types did not resist the test voltage after this cycle. The only configurations that withstood this test and continued to the next aging cycles were those composed of PAI + PAI/Sol–gel 2 + Filled thermoplastic resin and PAI + PAI/Sol–gel 3 + Filled thermoplastic resin. The one that presented a longer life is the PAI + PAI/Sol–gel 2 + Filled thermoplastic resin.

The thermal index has been estimated from the three temperature aging tests. The regression plot parameters were calculated for the wires which resisted longer at the lower-temperature 240 °C aging test. The three temperatures lifetimes allowed for the calculation of the coordinates and abscises, as presented in Table 4. 

The coefficients of the two-regression line have been calculated, and the temperature for a lifetime of 20,000 h and 2000 h have been estimated, as presented in Table 5. The thermal index obtained for each wire, also called “thermal class”, is the temperature determined for 20,000 h of lifetime. 

The Pearson coefficient is quite satisfactory, indicating that the experimental points are sufficiently aligned. This allows the interpretation of the difference between the EIS configuration’s thermal indexes. 

**Figure 15:**
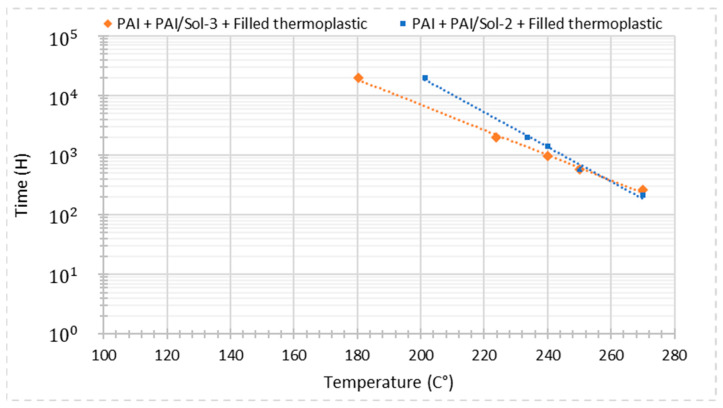
Experimental lifetime data plot for thermal aging.

The regression lines of both wires are plotted in the graphic presented in Figure 15. The inclination coefficient of the graphics resulted in this difference in the thermal index.

In this case, the latest test has defined the difference obtained between the thermal class of these enameled–extruded wires. A difference of 20 °C has been noticed between the two wires from this thermal index estimation. The Sol–gel type is the only difference between the two-insulation configuration. The Sol–gel 2 and 3 differs principally by the Mercaptan presence in the latter. This estimated difference in the thermal index indicates that the adhesion promoter influenced the reduction of the thermal index. The wire PAI + PAI/Sol–gel 2 + Filled thermoplastic corresponds to a 200 °C thermal class. A longer test should be carried out for both wires to obtain a more accurate estimation value of the thermal index. 

**Figure 16:**
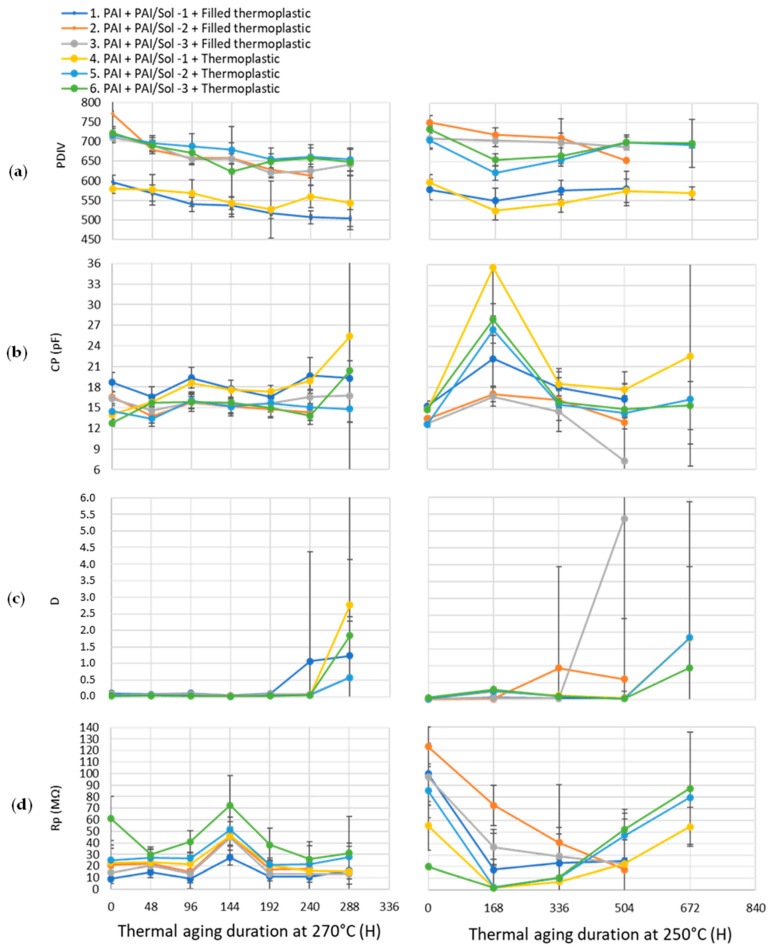
Dielectric parameters collected during aging at 270 °C and 250 °C: (**a**) PDIV, (**b**) parallel capacitance, (**c**) delta tangent, (**d**) parallel resistance.

The monitoring of the dielectric parameters between cycles was carried out at 270 °C and 250 °C, as shown in the curves of Figure 16. For both aging temperatures the dielectric parameters behave similarly. In both cases, the following can be concluded:

- PDIV values are decreasing. It is visible that the advantage in PDIV of wires 2, 3, 5, and 6 compared to the others is also due to the higher thickness of these wires. The partial discharges appear between 500 and 550 V for the wires composed of the PAI and Sol–gel 1 composite at the end of their life after aging at 270 °C and, at 550 V after aging at 250 °C. The decrease in this parameter is stronger during the aging at 270 °C compared to the aging at 250 °C.
- The parallel capacitance values show an increase for all wires, which represents the aging of this property for aging at 270 °C. For the wire composed of PAI/Sol–gel 1, the increase is even greater. For the curves of aging at 250 °C, the increase in this parameter is not as visible as for aging at 270 °C. Thus, as the decrease in the PDIV is slight, the increase in this parameter is almost not obvious.
- The dielectric loss factor values show a peak when most of the samples reach the end of their life. This phenomenon is visible for both aging at 270 °C and 250 °C.
- The parallel resistance values are very variable, especially at the end of the life cycle, which may be due to poor contact during measurement. For aging at 250 °C, the last measurement points show increases compared to the previous cycles. This is not physically representative since this parameter behaves inversely to the dielectric loss factor.

## 3. Conclusions

The insulation configurations have shown promising fulfilled electrical, thermal, and mechanical properties for new magnet wires, targeting their use in winding electrical machines. The wire which performed better on the thermal index parameter is the PAI + PAI/Sol–gel 2 + Filled thermoplastic (200 class), despite its electrical breakdown being lower than the minimum expected for its grade. The configurations PAI + PAI/Sol–gel 3 + Filled thermoplastic have performed satisfactorily in mechanical and electrical tests but with a lower thermal index (180 °C). 

The TGA analysis did not reveal a significant difference between Sol–gels 2 and 3 to indicate exactly which EIS wire could have the higher thermal index. Moreover, the silica content in the varnish from both sols was nearly comparable (16% and 18%) and, in a dry full EIS system, was estimated at 4.38 and 5.22% (at 700 °C). 

In the case of the EIS system with Sol–gel 1, the weight loss (from TGA analysis) at 406.73 °C and 509.15–546.39 °C appears more significant compared to the EIS system with Sol–gel 1 and Sol–gel 3. These characteristics are consistent with the thermal-aging results, which explain the EIS composed of Sol–gel 2 and 3 having an advantage over the Sol–gel-1 EIS on both tests: the GTA and thermal-aging tests. 

The thermal class parameter depends also on the other general properties of the EIS to support the proof voltage which determines its lifetime on thermal-aging tests (thermal degradation of the polymeric part the EIS systems, oxidation phenomena of the copper wires, wear resistance from partial discharge phenomena, etc.). The phenomenon of deterioration of dielectric properties during thermal aging must be more deeply investigated to understand the principal factors responsible for improving the thermal index of magnet wires. 

The hybrid coating obtained by the Sol–gel process in the new EIS systems has demonstrated a promising technique for enameling wire. The challenge is to incorporate an optimum concentration of silica Sol–gel in the full EIS system while keeping the mechanical properties (mainly flexibility) required for magnet wires. The extrusion layer (topcoat) helps improve the electrical, mechanical, and surface aspects of the wire. The use of mineral fillers in the thermoplastic resins improves thermal resistance during the aging tests and the tangent delta temperature peak of the insulation configuration. Furthermore, the primers (first and second thin layers) and extrusion contribute to an insulation process for magnet wires becoming eco-friendlier by reducing the energy consumption, and the amount of varnish and solvents (NMP, cresol) required for the conventional enameling process.

Moreover, an optimum silica content (in the first and second thin layers) combined with enameling process parameters (oven temperature and application speed during the enameling process) could increase the thermal resistance and therefore magnet wire properties. This work requires further investigation, which is the subject of future works.

## 4. Materials and Methods

The insulation configuration used for the magnet wires is presented in the first part of the section. The tests used for evaluation of EIS wires are described in the second part. 

### 4.1. EIS Application Process

**Figure 17:**
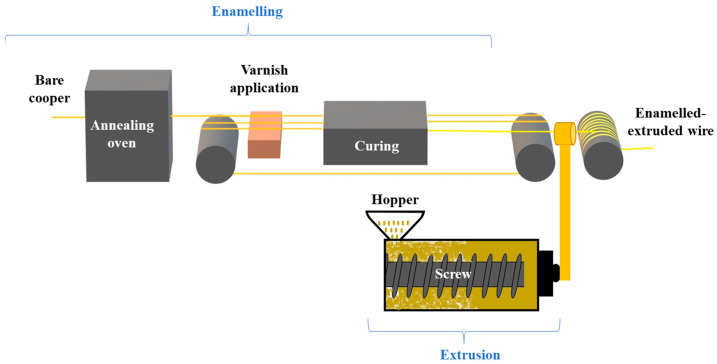
Enameling and extrusion process used for coating wires.

The enameling process associated with an extrusion has been used for the coating of wires. The coating occurs in the following steps, as illustrated in Figure 17:

- Enameling: The bare copper is introduced into an annealing oven, where it is cleaned and softened. Then, the varnish is applied to the copper-conductor surface, followed by a passage to the curing oven. The varnish application and the curing oven steps are executed many times until obtaining the desired diameter increase. The enameling speed is 16 m/min for the fabrication of the wires studied in this paper. The temperature is between 500 °C and 550 °C for both ovens, including both annealing and curing.
- Extrusion: This system is the most popular for thermoplastic forming [18]. The insulating material, usually thermoplastic polymers in pellet form, is fed through the hopper. The extruder contains a screw inside which is turned by a motor and gears (one or more depending on the extruder). The extruder is equipped with heating belts that help to plasticize the material. The molten material (also known as extrudate) is calibrated and cooled using auxiliary devices to calibrate pressure and cooling. After melting, the extrudate is pushed and moved according to the movement of the screw (in the direction of the die). The extrusion process temperatures are around 300 °C for the fabrication of the wires studied in this paper.

### 4.2. EIS Wire Configurations 

**Figure 18:**
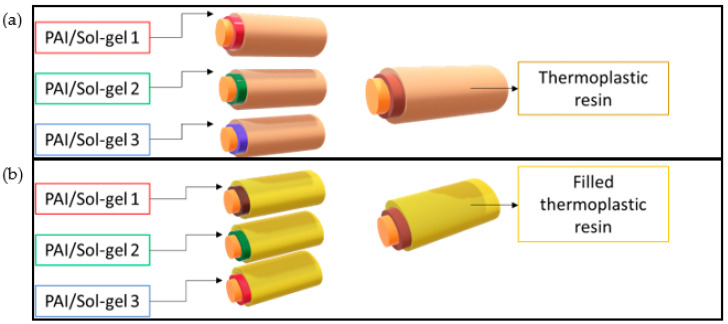
Insulated configurations using enamel-extruded technology overcoated by (**a**) neat thermoplastic resin, and (**b**) filled thermoplastic resin.

The EIS configurations are illustrated in Figure 18. The enameling coat is composed of three kinds of material:

- A thin layer of pure enameled PAI, given its positive influence on the properties studied in previous works [6].
- A second layer of silica–PAI enameled composites obtained from three different silica Sol–gel incorporated into PAI varnish.
- A third thick layer of extruded thermoplastic polymer. The same thermoplastic extruded resin used in previous works [6].

#### 4.2.1. Enamel Varnish Configurations

**Figure 19:**
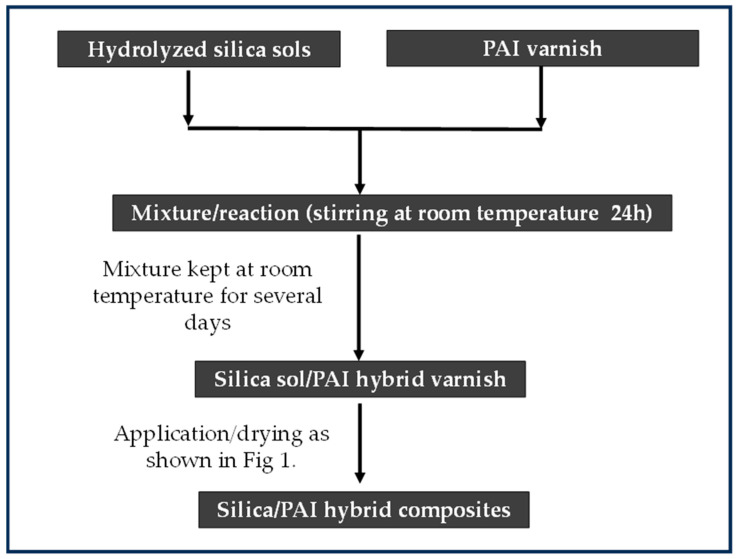
Scheme of enamel varnish configurations showing the reparation method of the enamel varnishes with different silica Sols/PAI (used as a primer for the second layer targeting 20 µm of wire diameter increasing).

The preparation of the enamel varnishes is shown in Figure 19. The first thin layer was obtained from pure PAI varnish (dry extract 20%), giving a dry thickness of about 1.5 µm (increased wire diameter by 3 µm). The second layer was obtained from hybrid composites (PAI–silica) prepared by the Sol–gel route. Silica/PAI hybrid varnishes were used, targeting a dry thickness of 10 µm (wire diameter increasing by about 20 µm).

Their principal characteristics are presented in Table 6. This hybrid (PAI–silica) varnish was prepared as follows:

- The silica sols were obtained separately. The silica Sol–gel named Sol–gel 1 was supplied by Materia Nova, ASBL, and the commercial Sol–gel 2, and Sol–gel 3 were supplied by Esix Surface Technologies Technology.
- The silica dry content (solid content after drying and gel formation) of the different sols was determined at 260 °C for 1 h in an oven under air. The solid content was estimated at 40%, 12%, and 14% for Sol–gel 1, Sol–gel 2 and Sol–gel 3, respectively.
- These different silica sols (in a liquid hydrolyzed state) were incorporated into PAI varnish, and kept at room temperature for several days. The established preparation process gives stable and homogeneous hybrid varnishes from Sol–gel 1, Sol–gel 2 and Sol–gel 3 when respecting the ratio silica/PAI (% solid to solid) of 30/70, 16/84 and 18/82, respectively.
- Finally, the hybrid silica–PAI varnishes were used in the enameling process for obtaining the second layer schemed above (before the extrusion layer topcoat). Sol–gel 2 and 3 were limited in miscibility, so they were tested at lower concentrations not exceeding 20%. The estimation of silica content in the primer layer and the filler into the thermoplastic topcoat was determined experimentally from TGA analysis (residual par) in the EIS systems with unfilled thermoplastic and filled thermoplastic.
- The estimation of silica (SiO_2_) in the dried EIS systems was determined from thermogravimetric analysis (TGA), assuming that the residue value obtained at 700–1000 °C corresponds to the mineral part (SiO_2_) for the unfilled thermoplastic EIS coatings (PAI/Sol–gel–PAI + thermoplastic). In the case of filled thermoplastic EIS systems (PAI/Sol–gel–PAI and filled thermoplastic), the remaining mineral part (residue) was assumed to correspond to the sum of silica (from hybrid varnish PAI–Sols), and the mineral fillers from the filled thermoplastic extruded polymer. These different configurations are reported in Table 6.

#### 4.2.2. Extruded Resin Configuration

The extruded resins are the same thermoplastic polymer. This polymer had been filled with mineral micro-fillers already used in previous works [6]. The general characteristics are presented in Table 7.

The thicknesses are variable as the different enameled layer products present variations in dry contents. This causes variations between the enameled layers, at the time of application, despite the number of passes (varnish applications) being the same. The insulation coat size targets are as follows:

- 5% of the whole thickness by one pass of enameled PAI.
- 20% of the whole thickness obtained by the solution from the Sol–gel process mixed with enameled PAI.
- 75% of a resin coated by the extrusion process.

The IEC 60317-0-1 [15] specifies grades (insulation thickness) for several wire conductor sizes; the wires studied in this work are 0.95 mm. The dimensions closest to this case study are the 0.9 mm and 1 mm conductor diameters. The grades listed in Table 7 are defined for a 0.9 conductor size. According to the insulation thickness obtained by SEM characterization in Section 2.1.1, the filled extruded wire is classed as grade 3, and the non-filled extruded wire is classed as grade 2. 

### 4.3. Evaluation Methods and Tests 

All the insulated wire characterizations have been made according to IEC standards [15,19,20,21,22,23,24,25,26,27,28,29]. 

#### 4.3.1. Basic Insulated Wire Properties

The basic properties collected are the mechanical and electrical properties. These are some of the essential characteristics of magnet wires.

**Figure 20:**
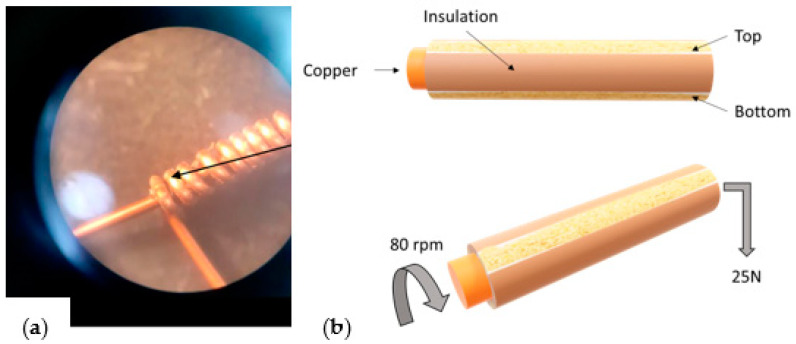
Mechanical tests: (**a**) flexibility test example, and (**b**) peel test illustration.

The mechanical properties are characterized following the IEC 60 851-3 standards for enameled wires [22]:

- Flexibility: This test consists of winding the wire around its diameter. The verification of the insulating surface is performed once these turns are made. The sample is observed with a binocular (6× zoom) and classed OK if it resists the test without presenting any cracks, and KO if the sample presents cracks after these mechanical constraints.
- Adhesion: The adhesion is verified by two tests: the snap test, and the peel test. The first one is made in a 25 cm of wire length which is stretched until it breaks, and if it caused cracks or the stripped part is visible, the sample adhesion is considered bad (KO); otherwise, it is considered OK. For the peel test, 50 cm of the wires were extended, and a 25 N load was placed at one of its extremities, as illustrated in Figure 20. The top and bottom parts of the insulation were removed. At these conditions a motor placed in the other extremity makes the wire rotate at a speed of 80 rpm, and the number of rotations to reach the loss of adhesion is counted and noted.

The breakdown voltage is the electrical parameter collected for the proposed insulated wires. This test is carried out for five samples per insulated wire as per the IEC 60851-5 standard recommendations [19]. The samples used are twisted pairs. The test voltage is applied between the two conductors of the specimens at a rate of 100 V/s for breakdown voltage from 500 V up to 2500 V, or 500 V/s for higher breakdown voltage. In this case, the voltage rate is 500 V/s. The expectation is to obtain at least the minimum voltage defined for insulated wires of grades two and three, being, respectively 5000 V and 7600 V [15]. 

#### 4.3.2. Thermal Evaluation Methods and Morphology Observation

The evaluation consists of two groups of tests, 1 and 2 described below:

1. TGA, SEM and FTIR analyses.
  - Thermal evaluation using thermogravimetry analysis (TGA): The thermal stability of the studied materials was evaluated by TGA analysis under air atmosphere using TGA Mettler Toledo (Columbus Instruments, Columbus, OH, USA) by heating the sample from room temperate (RT) to 1000 °C in a 70 µL Saphir sample holder with a heating ramp of 10 °C/min.
  - Surface and cross-section morphology evaluation using SEM: The analyses of the top surface morphology and cross-section were collated by SEM analysis available at Materia Nova, ASBL (SEM, Hitachi, SU8020, Tokyo, Japan). The equipment is coupled with an energy-dispersive X-Ray spectrometer analyzer (EDX, Thermo Scentific Noran System 7, Madison, WI, USA), allowing for quantitative chemical element estimation.
  - The FTIR analyses were carried out on the magnet wires for the different EIS configurations. The FTIR measurement was recorded with IRTracer-100 (Shimadzu Co., Kyoto, Japan) within the 4000–550 cm^−1^ wavenumber range.
2. The thermal tests for insulated wires destined for winding application were as follows: cut through, tangent delta curve, thermal-aging tests, and thermal index estimation.
  - The cut-through characteristic of the insulated wire is obtained by ASTM D1676-03 [30]. This test consists of measuring the temperature required to deform the material sufficiently to allow electrical contact. The samples were two pieces of wires, with their extremities stripped. A load was placed perpendicular to the crossing of the two wires to ensure physical contact between them. The point of crossing was heated. Once the device test was launched, a temperature was applied to the point of contact between the two round wires and increased until the maximum current that characterizes the fault was detected. The latter corresponded to 0.1 A at 115 V (50 Hz).
  - The tangent delta versus temperature characteristics of each wire were collected according to IEC 62631-1 [23] and ASTM D 150-98 [31]. This property identifies the appropriate curing range of a given film when all physical, chemical, thermal, and electrical properties of the wire are acceptable. The setup measurement consists of a computer connected to a vented oven where the sample is placed for measurement. Before inserting the sample into the oven, a graphite solution is applied to the insulation surface of the wire and the wire is then dried. The area covered by the graphite will be in contact with the electrodes of the measuring device inside the oven. The value of the dissipation factor is collected at a frequency of 1000 Hz while the temperature of the oven increases from 50 to 350 °C. One sample for each insulated wire studied is measured.
  - The thermal-aging tests are part of the protocol for determining the thermal index of the insulated wire. In this test, groups of 11 twisted pair samples were aged under high temperatures at several cycles. The temperature exposition and the duration of cycles are based on the recommendation of the standards IEC 60 216-1 [26]. For each cycle of exposition, the proof voltage was applied, and its amplitude was chosen according to the thickness of the insulation to the IEC 60172 [21,29]. The lifetime was determined when more than 50% of the samples failed in the proof voltage. Additional measurements between cycles have been collected of the eleven samples per EIS for two of the aging tests, with the Agilent 4294A precision “LCR meter” impedance analyzer based on the measurement methods defined by IEC 62631-1 [23] and ASTM D 150-98 [31]. The parallel capacitance (Cp), parallel resistance (Rp), and delta tangent value (Tan δ) were measured. The measurement frequency was chosen according to the accuracy given in the catalog (10 kHz-0.08% accuracy). The necessary corrections (open circuits and short circuits) were made before the measurements to obtain the most representative values of the sample. The value of the Partial Discharge of Inception Voltage (PDIV) was also collected using a measurement device designed according to the IEC 60270 [28].

Those additional measurements allow the visualization of the damage of the properties over the aging time until the end of life, and to compare with other insulating configurations submitted to the same stress.

- The thermal index is estimated for the wires which held enough cycles during thermal aging at the three temperatures. The thermal index estimation is determined by the IEC 60216-3 standard [24]. The steps of the analysis of the experimentation data are the following. The experimental data were transformed into x and y values, based on Equations (1) and (2).

(1) $$x_{i}=\frac{1}{T+273},$$

(2) $$y_{ij}=\log t,$$

where:

T, the temperature of exposition in °C;

t, the lifetime in hours, which is determined by the total of aging cycles supported, subtracting the half duration of the last cycle.

The mean x and y values are calculated by Equations (3) and (4) where k is the number of temperature expositions.

(3) $$\bar{x}=\sum \frac{x_{i}}{k},$$

(4) $$\bar{y}=\sum \frac{y_{i}}{k},$$

The coefficients of the regression equation are given by Equations (5) and (6). The calculation of thermal index corresponds to the lifetime of twenty-thousand hours (IT= 20,000 h). It is calculated by X in Equation (7), with Y=log20,000.

(5) $$b=\frac{\sum x_{i}\bar{y_{i}}-k\bar{x}\bar{y}}{\sum x_{i}{}^{2}-k\bar{x}2},$$

(6) $$a=\bar{y}-b\bar{x},$$

(7) $$X=\frac{Y-a}{b},$$

## Figures and Tables

**Table 1:** TGA parameter obtained from the different EIS systems.

	* T_d_ °C/(% Weight Loss)	T_d_ °C/(% Weight Loss)	OTD ** °C (Maximum Weight Loss)	Residue at 950 °C
PAI + PAI/Sol–gel 1 + Thermoplastic	406.73 (3.04%)	509.15/546.39 (16.37/13.11%)	608.18 (40.99%)	5.68%
PAI + PAI/Sol–gel 1 + Filled thermoplastic	409.27 (2.51%)	508.30 (5.36%)	589.55 (42.81%)	27.22%
PAI + PAI/Sol–gel 2 + thermoplastic	457.51 (4.66%)	509.99 (13.94%)	603.10 (46.84%)	4.38%
PAI + PAI/Sol–gel 2 + Filled thermoplastic	456.67 (4.58%)	509.99 (11.16%)	604.79 (40.29%)	24.35%
PAI + PAI/Sol–gel 3 + thermoplastic	452.44 (5.81%)	508.30 (13.81%)	601.40 (44.64%)	5.22%
PAI + PAI/Sol–gel 3 + Filled thermoplastic	456.67 (3.85%)	510.84/546.39 (10.52/9.78%)	609.02 (29.63%)	23.32%

* Td presents the decomposition steps related to pyrolytic fragmentation. ** OTD presents the temperature (°C) at which the maximum degradation is observed.

**Table 2:** Mechanical and electrical properties.

Samples	PAI + PAI/Sol–gel 1 + Thermoplastic	PAI + PAI/Sol–gel 1 + Filled Thermoplastic	PAI + PAI/Sol–gel 2 + Thermoplastic	PAI + PAI/Sol–gel 2 + Filled Thermoplastic	PAI + PAI/Sol–gel 3 + Thermoplastic	PAI + PAI/Sol–gel 3 + Filled Thermoplastic
Snap test	OK	OK	OK	OK	OK	OK
Peel test (turns)	140	145	140	160	146	135
Flexibility	KO 1D	KO 1D	OK 1D	OK * 1D	OK 1D	OK * 1D
Breakdown voltage (kV)	7	7.5	9	5	8.7	8
Over diameter (µm)	58	110	68	90	84	103

* Color changes during the flexibility test.

**Table 3:** Thermal test results of the insulated wires.

Samples	PAI + PAI/Sol–gel 1 + Thermoplastic	PAI + PAI/Sol–gel 1 + Filled Thermoplastic	PAI + PAI/Sol–gel 2 + Thermoplastic	PAI + PAI/Sol–gel 2 + Filled Thermoplastic	PAI + PAI/Sol–gel 3 + Thermoplastic	PAI + PAI/Sol–gel 3 + Filled Thermoplastic
Cut-through temperature (°C)	509	511	480	433	472	472
Tangent delta temperature (°C)	103.5; 272.9; 347.2	100.9; 218.0; 260.8; 293.2	104.4; 264.9	102.4; 265.8; 274.3; 291.8	99.2; 259.9	96.5; 275.7; 283.5
Lifetime at 270 °C (H)	264	264	264	216	264	264
Lifetime at 250 °C (H)	420	588	420	588	420	588
Lifetime at 240 °C (H)	<720	<720	<720	1440	<720	960

**Table 4:** Parameters of enameled–extruded wires lifetime under thermal aging.

Temperatures		PAI + PAI/Sol–gel 2 + Filled Thermoplastic		PAI + PAI/Sol–gel 3 + Filled Thermoplastic	
T (°C)	X=1TK	*Y* = *a* + *BX*	Lifetime (h)	*Y* = *a* + *BX*	Lifetime (h)
270	0.00184	3.39 × 10^−6^	216	3.39 × 10^−6^	264
250	0.00191	3.66 × 10^−6^	588	3.65 × 10^−6^	588
240	0.00194	3.80 × 10^−6^	1440	3.79 × 10^−6^	960

**Table 5:** Coefficient of enameled–extruded wires lifetime versus temperature regression graphic under thermal aging.

Wire	Number of Temperature Aging	*a*	*B*	Pearson Coefficient	T °C (20,000 h)	T °C (2000 h)
PAI + PAI/Sol–gel 2 + Filled thermoplastic	3	−11.4	7458.4	0.98	201.3	233.5
PAI + PAI/Sol–gel 3 + Filled thermoplastic	3	−7.1	5171.1	0.99	180.3	223.9

**Table 6:** Enamel varnish configurations with different silica sols.

Solutions	Sol–gel 1	Sol–gel 2	Sol–gel 3
Solid content of the silica Sol–gel (1 h at 260 °C)	40%	12%	14%
Estimated silica solid content into PAI (solid to solid)	30%	16%	18%
* Estimated silica solid content into the full unfilled thermoplastic EIS system	5.68%	4.38%	5.22%
* Estimated “silica + filler” solid content into the full EIS filled thermoplastic system (700 °C)	27.22%	24.37%	23.32%
* Estimated filler loading (into the filled thermoplastic)	21.51%	20%	18.10%

* Estimation from TGA analysis calculated at a temperature of 700 °C.

**Table 7:** General characteristics of Sol–gel-enameled overcoat by thermoplastic resin.

Samples Id	PAI + PAI/Sol–gel 1 + Thermoplastic	PAI + PAI/Sol–gel 1 + Filled Thermoplastic	PAI + PAI/Sol–gel 2 + Thermoplastic	PAI + PAI/Sol–gel 2 + Filled Thermoplastic	PAI + PAI/Sol–gel 3 + Thermoplastic	PAI + PAI/Sol–gel 3 + Filled Thermoplastic
Insulating configuration (Figure 18)	(a)	(b)	(a)	(b)	(a)	(b)
First enamel coat	PAI	PAI	PAI	PAI	PAI	PAI
Second enamel coat	Sol–gel 1 (30%)/PAI (70%)	Sol–gel 1 (30%)/PAI (70%)	Sol–gel 2 (16%)/PAI (84%)	Sol–gel 2 (16%)/PAI (84%)	Sol–gel 3 (18%)/PAI (82%)	Sol–gel 3 (18%)/PAI (82%)
Extruded coat	Thermoplastic	Filled thermoplastic	Thermoplastic	Filled thermoplastic	Thermoplastic	Filled thermoplastic
Over diameter (µm)	58	110	68	90	84	103
Grade	2	3	2	3	2	3

## Data Availability

The data are not publicly available due to privacy considerations.
